# Artificial intelligence and medical futility

**DOI:** 10.1186/s12911-026-03635-6

**Published:** 2026-06-17

**Authors:** Sinead Prince, Dominic J. C. Wilkinson, G. Owen Schaefer, Finn Lip, Brian D. Earp, Julian Savulescu

**Affiliations:** 1 Centre for Biomedical Ethics, Yong Loo Lin School of Medicine, National University of Singapore, 10 Medical Drive, #02-03 MD11, Singapore, 117597 Singapore; 2 Uehiro Oxford Institute, University of Oxford, Suite 1, Littlegate House, 16-17 St Ebbe’s Street, Oxford, OX1 1PT UK; 3 Murdoch Children’s Research Institute, Melbourne, Australia; 4 School of Biomedical Sciences, University of Western Australia, Perth, Australia

**Keywords:** Medicine, End-of-life, Futility, Benefits, Bioethics, Artificial intelligence, Justice, Preferences, Decision-making

## Abstract

**Background:**

Determining when medical treatment is futile is conceptually, empirically, and ethically disputed. This creates challenges for patients, families, doctors, healthcare, and legal systems. Amid such disputes, there is still a practical need to resolve futility questions in individual cases. Can artificial intelligence (AI) be ethically and effectively used to help with such decisions?

**Methods:**

We adopted a critical narrative review method. We first surveyed existing scholarship on the ethics of medical futility to identify the main positions in ongoing futility debates. We then surveyed existing or potential medical AI devices to identify whether such tools might be used to help address these debates or their practical upshots. Finally, we philosophically analysed whether such tools ought to be used, identifying and weighing reasons using analytic argument, applying relevant ethical concepts, and philosophical principles, such as respect for autonomy, beneficence, non-maleficence, and justice.

**Results:**

We found that there are some key challenges or risks in using AI for such purposes, for example, using AI may exacerbate the problem of self-fulfilling prophecies in futility determination by absorbing predicted outcomes as data. Nevertheless, under certain conditions, medical AI could ethically be used to prevent or help resolve futility disputes in healthcare decision-making.

**Conclusions:**

AI could ethically contribute to preventing or resolving futility disputes depending on how AI is integrated and regulated in end-of-life care.

## Background

Sometimes, in medicine, a judgment must be made that further treatment is unlikely to be effective or will not be worth pursuing. Treatment in these cases is said to be futile. This concept is relevant to all medical care, but in intensive care, a declaration of futility has significant consequences: withdrawing life-sustaining treatment foreseeably results in patient death.

The stakes of futility judgments can be high. And yet, there are well-known and persistent disagreements about the concept of futility (what it means for treatment to be futile), the practice of making futility judgments (how to determine whether a given treatment is futile), and what to do when the patient or family disagrees with a judgment that treatment is (or less commonly, is not) futile.

Instead of defining futility clearly, some hospitals adopt a procedural approach to determining futility: they use a certain process or procedure thought to provide legitimacy to such judgments, such as asking a special committee of healthcare professionals to decide on specific cases. If they decide that treatment is futile, then, according to this method, it is futile. This, in turn, is thought to justify withdrawing life-sustaining treatment, even over the objections of the patient or their family. But this approach has also been criticized. Some argue it just shifts the decision from one set of doctors to another set of doctors without addressing the underlying problem [1, 2]. Others have expressed concern that there is typically no outside review in such cases to make sure that the resulting decisions are fair [3]. Without a substantive, implementable, and widely agreed-upon account of futility, there may be no way to make determinations of futility in a way that is consistent, transparent, and just.

This has several negative downstream consequences. For example, determining futility and rationing without a clear concept or set of criteria causes significant moral distress and burnout within practitioners [4–6]. Providing care that is plausibly futile (on some conceptions) in the context of a resource-strapped healthcare system is also morally problematic.^1^  

We need better solutions to these disputes, and as technological advances continue to extend life-sustaining capacity, this need will only intensify. In this paper, we consider whether artificial intelligence (AI) could be ethically and effectively used to help with decisions around medical futility in practice, notwithstanding such ongoing disagreements.

Following the critical narrative review method [10], we first conduct an analysis of the major existing concepts of futility and possible ways of resolving disputes between families/patients and doctors/hospitals over when treatment is futile. Second, in what follows, we propose a way forward: we consider whether AI might assist in determining whether treatment is futile according to each major concept. Last, we philosophically analyse whether AI (if suitably trained and developed for the purpose) should be used to assist with decisions about futility. To do so, we identify and weigh reasons using analytic argument, apply relevant ethical concepts, and philosophical principles, such as respect for autonomy, beneficence, non-maleficence, and justice [11]. For instance, we consider how using biased data or algorithms might make discriminatory decisions about treatment cost-effectiveness, and how AI tools might exacerbate concerns that current decisions about treatment futility are opaque and procedurally unfair. We will argue that there are ways to prevent these concerns from arising and that these concerns might be outweighed by the benefits of using AI. We conclude that AI has a significant role to play in reducing the costs and burdens of determining when patient treatment becomes futile, but its design and use ought to be regulated.

## Methods

For this project, we adopted a narrative review methodology, specifically, a critical review process [10]. Narrative reviews are non-systematic, subjective, and interpretivist; they survey a field of literature or theories, particularly well-developed fields, to develop new ways to advance the field [10]. Critical narrative reviews are a non-comprehensive form of narrative review that includes both critical analysis of the existing literature and some form of conceptual or theoretical innovation [12]. Such reviews are non-comprehensive in that they do not seek to survey everything that has been said in the field of inquiry, but only that which is most relevant and significant [13].

Following this method, we first undertook a non-systematic literature review of medical and ethical literature for papers addressing conceptual or definitional issues with the term “futility”, proposing criteria for its evaluation, or analysing ethical considerations. Further papers were identified from the reference lists of reviewed articles. This process was repeated until saturation, that is, until no new concepts or ethical issues relating to medical futility had been raised, and each new article had heavily referenced already reviewed articles.

Once the main conceptual frameworks for understanding medical futility had been identified, we considered, and in some cases researched, existing AI tools that could be used to achieve more consistent, transparent, and reliable determinations of medical futility, according to each framework.

We then considered whether we should use AI in such ways. This first involved another non-systematic literature review of existing ethical issues pertaining to the use of AI in healthcare, supplemented by targeted searches for literature addressing AI in end-of-life care contexts. We then philosophically analysed the ethics of using AI within this medical context: we identified and weighed reasons using analytic argument, applied relevant ethical concepts, and philosophical principles, such as respect for autonomy, beneficence, non-maleficence, and justice [11]. This ethical analysis generated some guidance or recommendations for future research and implementation of AI in determinations of medical futility.

## When is treatment futile?

Futility has been defined in various ways, with different proposed synonyms and subtypes.^2^ Broadly, however, the literature can be divided into quantitative and qualitative conceptual frameworks.

A quantitative conception of futility holds that a treatment is futile (i.e., should not be provided) when it has a low probability (or magnitude) of net benefit.^3^ But this presents two main problems: first, how do we know what the probability or magnitude of net benefit is for any given treatment (an epistemological problem); and second, how should we decide what probability or magnitude is too low to justify ongoing treatment (the normative problem). On the one hand, it might seem that these problems could be solved by drawing a statistical threshold for “futility” based on objective clinical indicators (e.g., disease severity and survival thresholds). On the other hand, such an approach may not be well-justified due to the problem of self-fulfilling prophecies: if declared futile, then treatment is withdrawn, and this elevates mortality rates for those illnesses [3, 19]. Moreover, such thresholds (e.g., 1%, 0.1%, 0.01% chance of full recovery) are also arbitrary, and patients may reasonably seek interventions with a very low chance of success when the alternative is certain death [20].

Alternatively, treatment may be judged qualitatively futile because of an anticipated low quality of life. For example, providing surgery for bowel obstruction in a permanently unconscious patient may be judged futile because (even if the operation is successful), the patient will remain in a state of complete lack of awareness. However, these qualitative judgements raise several questions: for whom the anticipated quality of life is acceptable, and what counts as an acceptable quality of life [3]. Answering such questions becomes even harder when futility disputes arise for patients who lack capacity. In these cases, surrogates must determine the patients’ preferences about the anticipated quality of life from the treatment. And yet, research suggests families and surrogates fail to accurately identify the patient’s actual preferences in one-third to one-half of cases [21–23]. Research also shows that families often seek to prolong the life of their loved one, for reasons including unreasonable expectations of improvement, religious beliefs, or guilt for ending the patient’s life [24]; even if prolonging treatment would be inconsistent with the patient’s own preferences. Finally, Savulescu [20] argues that we cannot guarantee treatment is ever going to be 100% ineffective and as such, we should presume that treatment will generally be in the patient’s best interest given the guarantee of worse outcomes in the alternative of withdrawing treatment. Disputes about the extent of the benefit of the treatment can therefore arise when using the qualitative concept of futility between patients and doctors.

Some authors acknowledge these approaches, but argue that at the core of determining when to withdraw treatment is not whether the treatment is futile, but whether treatment is fair and just to provide in the circumstances [19, 20]. They argue that the strongest reason to withdraw treatment is that it is not cost-effective, and that fairness to other patients must also be considered through distributive justice principles, such as preventing discrimination and bias. However, doctors do not always clearly distinguish between futility, in this sense, and rationing [25]; they sometimes inform patients that treatment is futile (so that the patient believes there is nothing that can be done), but the core reason is that treatment is just too expensive or resource intensive [4]. Whether or when treatment may be withheld to ration remains contested, and we acknowledge that it is important to separate rationing decisions from other reasons not to provide treatment.

For the purposes of this narrative review, we need not commit to a particular use of the concept of futility. Arguably, there can be a range of justified uses of the concept [26]. However, at a high level of generality, we note that the concept of futility is used to identify situations in which some purported benefit or aim is not realistically achievable. We also accept that it is reasonable for different parties to pursue different aims or benefits. Doctors, for instance, might be justified in valuing or aiming for medical benefits and identifying situations in which these goals are extremely unlikely to be met. Patients might also be justified in identifying when medical treatment would potentially be beneficial to their life as a whole. The key ethical question in futility disputes relates to how to arbitrate when parties value conflicting aims or benefits, or hold conflicting views about whether a given treatment is sufficiently likely to achieve a particular benefit.

## Approaches to resolving disputes

Several main ways of arbitrating such disputes have been proposed in the literature. The first way purports to maximize patient autonomy; the second places certain “reasonableness” constraints on patient autonomy; and the third emphasizes the process of resolving the dispute fairly, rather than determining a particular solution. We will briefly summarise these approaches to disputes.

Some authors argue that patient autonomy ought to hold the most weight in determining whether treatment is futile in disputes. Given that medicine is – and as they believe, should be – increasingly patient-oriented and prioritising patient autonomy, only the patient (or their surrogate) can determine whether treatment is futile as measured against the patient’s personal goals for treatment [3]. As such, when instances of irreconcilable disagreement over values arise within pluralist healthcare systems, the patient’s autonomous choice ought to be prioritized (or in the case of a patient lacking capacity, a surrogate determination of what the patient would have chosen) [19]. Simply because the doctor may not share the patient’s values in relation to their life goals, does not itself give reason to privilege the doctor’s view that medical values ought to take precedence [1]. On the patient-based approach, two key questions arise. First, whether the patient’s preferences are actually likely to be satisfied by the treatment, and second, of patients lacking capacity, whether surrogate decision-makers are correctly transmitting and applying the views of the patient.

Other authors argue that respecting patient autonomy is limited by reasonability constraints [19]: treatment ought to only be provided to the patient when its value could not be reasonably disputed. For example, instances of irrationality (such as a complete mismatch between treatment and problem, e.g., prescribing antibiotics for a viral infection), would render the patient or family’s desires unreasonable [5]. Similarly, when the treatment is highly likely to cause significant harm with a very small chance of success [27], such as when the side effects of a treatment would render the patient considerably worse off than if left untreated, it may be unreasonable to provide it. Determining whether treatment is unreasonable can be difficult to pinpoint; however, Wilkinson and Savulescu [19] suggest that surveying public preferences in determining futility could help assist doctors and hospitals make publicly justifiable decisions to withdraw treatment. Nevertheless, determining whether a patient’s request is unreasonable might be normatively challenging and time-consuming.

Some literature has moved towards preventative and responsive means to deal with futile cases in practice, and this is reflected in changes in terminology, e.g., labelling futile treatment as ‘disputed treatment’. For example, warning patients and families of expected outcomes in advance, ensuring decision-making is transparently communicated to patients and families, providing clear guidelines for dispute resolution if a disagreement about treatment arises, and recommending advanced healthcare directives are undertaken [5, 26, 28–30]. The American Medical Association recommends a five-step dispute resolution process, and if no solution is found, referral to an ethics committee, transfer of care to another institution, and finally, cessation of treatment [17]. Some legislated procedures require doctors to seek ethics committee review to withdraw allegedly futile treatment, but these have been criticized for simply deferring decision-making to even more doctors through a committee [1], lacking external judicial review and fairness, and failing to provide criteria to base decisions in consistent and transparent ways. Some jurisdictions, e.g., Texas, permit the withdrawal of futile treatment as determined by a doctor if the doctor has consulted an ethics committee [3]. Other jurisdictions, e.g., Canada, prohibit the unilateral withdrawal of potentially life-sustaining treatment, and require physicians to undertake conflict resolution with substitute decision makers; authority ultimately rests with an independent board or the substitute-decision maker [28].

The status quo is, thus, that different jurisdictions – and even different institutions or healthcare teams – adopt different concepts of futility, with no overarching agreement on what the correct concept or application criteria should be. Thus, it might seem, well, futile to introduce artificial intelligence into this cacophony: how can AI help with futility determinations if we cannot even agree what the right concept of futility is? But under current conditions, futility decisions of some sort are already being made and will continue to be made whether or not AI is involved. Thus, the starting point for our analysis is to ask how AI, given a certain approach to futility as actually applied within certain contexts, could help to improve decision-making based on the aims of the given approach? This way, we need not resolve debates concerning what the overall correct approach is or should be, and can instead focus on whether, or how, AI technologies can improve on the status quo based on a plurality of defensible approaches.

## How could AI help?

One common feature across the main approaches to determining futility is the integration or balancing of a large number of different factors (albeit, as noted, often toward different particular ends). However, this integration or balancing is often done in a highly subjective, non-standardised, and opaque way. Existing literature on the capabilities of AI gives us reason to believe that AI could broadly help in these cases due to its ability to algorithmize multiple factors derived from big data. This means that AI could provide inputs relevant to futility determinations, according to each of the main approaches, in consistent, empirically determined, and normatively justified ways. Having surveyed some emerging literature, we will show how AI might be used to inform each of these main approaches.

### Qualitative futility

The first approach we will consider is how AI might assist in determining the patient’s preference for treatment, namely, determining futility according to a qualitative definition. One example is online dispute resolution tools that use AI for optimizing and identifying discrepancies between disputing parties, such as factual disagreements [31]. These AI tools might assist in clarifying whether the treatment is futile according to the patient’s goals of treatment. For example, an AI-enhanced online platform might use data from the patient’s medical history and prognosis, the patient’s goals of care, and the extent of medical capacity to achieve these goals, to produce a table of differences and common positions, to streamline and identify the key aspects of debate, and to identify factual errors and irrational or unrealistic expectations: that is, to ensure all parties agree on a common set of facts or rational beliefs. This would ensure that doctors do not overstate claims that are uncertain, and patients or families are not mistaken about the likelihood of success. Ensuring mutual understanding as to the patient’s goals of care and the limits of medicine, situating this information within the context of the patient’s prognosis and medical history, and identifying the exact points of disagreement, might reduce disputes that arise due to empirical misunderstandings [3].

However, in intensive care (or when patients are acutely unwell), most patients do not have advance directives to indicate their preferences and do not always have the capacity to state their preferences [30]. Consequently, such patients often depend on substitute decision-makers to determine their preferences. However, research shows that surrogates fail to accurately identify the patient’s preferences in one-third to one-half of cases [21, 22]. Furthermore, for patients who do have advance directives in place, some research indicates only 70% of patients’ preferences for end-of-life care are stable over time [32]. Could there still be a role for AI in such cases?

In instances when the patient lacks capacity or an explicit advanced directive, AI might assist families and substitute decision-makers in identifying or predicting patient preferences during disputes over treatment. Current research shows that AI algorithms can predict patient preferences using the patient’s demographic data to assist doctors in formulating clinical treatment plans [33] and to assist healthcare systems in optimizing resource allocation and healthcare quality [34]. However, these models have been criticized for failing to incorporate individual patient values and preferences into the population-level data; such models make predictions based on what other people of similar demographics generally prefer rather than actual patient preferences [35]. These models might be statistically accurate but arguably fail to respect the patient’s autonomy to exercise their own decision.

As such, Earp et al. [36, 37] have recently proposed a “personalized patient preference predictor”: a hypothetical personalized version of the original patient preference predictor that has been trained on information or data describing, pertaining to, or produced by the patient. This could be used to predict the patient’s preferences for treatments and how treatment decisions are made. It could include patient preferences about how much weight to place on their own wishes versus the recommendations of health professionals or the views of family members. It could (at least sometimes) indicate how the patient would view the role of AI predictions in decisions.^4^ Such models would only apply when the patient has lost capacity and in which surrogates and clinicians are already making predictions [36]. Using such a personalized predictive model to more accurately represent the patients’ preferences in instances of dispute will satisfy, as best we can, the importance of allowing the patient to determine for themselves when treatment would be sufficiently beneficial to pursue. This tool could therefore be useful to physicians or substitute decision-makers who are required to determine and follow the patient’s preferences as to the pursuit or withdrawal of futile treatment.

One limitation to this application is suggested by research on patient preference stability about their end-of-life care. In one systematic review of the literature, Auriemma, Nguyen, and Bronheim [32] identified that whilst most patients experience preference stability over time and across changes in health status, a significant minority of patients experience unexpected changes. Limitations may therefore arise if the AI model is only capable of predicting the patient’s preferences about future health conditions, rather than the preferences of the future person. However, this is a limitation of any prediction, AI or human, about a patient’s preferences in the absence of their capacity to express them. Indeed, surrogate decision-makers are no more reliable than chance at predicting patient preferences, and according to the above review, approximately 30% of patients make unpredictable changes to their own preferences. In contrast, AI may be an overall more reliable, even if not perfect, means of determining patient preferences over existing surrogate models. Perhaps AI may not be able to predict future changes in preferences, but research is yet to show whether it does a worse, or even equally accurate, job at predicting patient preferences compared with surrogates’ predictions. The most important point is not that we should give up attempting to predict the patient’s own future preferences but rather that such predictions should be a component, but not solely determinative, of treatment decisions. Considerations of the patient’s future health and well-being (or best interests) objectively considered should also play some role, and is perhaps most salient when the predicted preference is to limit life-sustaining treatment. Here fallibility is especially consequential and consideration of adaptation or preferences and objective considerations relating to well-being play a role, such as quality of future relationships with friends and family.

### Quantitative futility

The second approach defines futility according to medical factors. According to this approach, the expertise of doctors in understanding the nature, effect, and limits of medicine justifies their authority to determine when the use of a particular treatment would do more physiological harm than good to the patient’s health. Although, historically, research has failed to establish a threshold of when treatment becomes medically futile (because this concept is, after all, a value judgement), machine learning could assist in providing certainty to the factors on which doctors base their calculations. AI and machine learning have been used to make accurate and timely prognostications of various health outcomes. For example, machine learning models have potential to accurately predict defibrillation success after cardiac arrest [38]; deep learning models can accurately predict palliative care prognostics after just 48 h of data collection [39]; explainable machine learning has accurately predicted 30-day, 90-day, and 1-year mortality rates in critically ill patients under mechanical ventilation [40]; and machine learning models have been able to successfully predict patient five-year survival rates with breast cancer to better equip doctors to avoid unnecessary and harmful therapy [41]. To a much greater degree than conventional prediction models, machine learning can also make personalized predictions of patient prognosis and response to treatment by incorporating a range of different individual-patient-specific factors [42].

The increased specificity and accuracy of such predictions is relevant to disputes over the benefits of treatment; it could help doctors, clinical ethicists, and hospital committees not only make more reliable and evidence-based predictions but also potentially distinguish between reasonable and unreasonable disagreements. If there is an agreed prognostic threshold for when professionals are justified in unilaterally withholding treatment, AI predictions would provide a more robust basis for decisions. This is not to say that machine learning can itself determine what would constitute an ethically acceptable threshold of benefit below which treatment may be deemed futile (e.g., X% chance of surviving to hospital discharge, or Y% chance of recovery from a state of unconsciousness and unawareness). We only suggest that increased certainty in prognostics allows physicians to properly weigh the benefits and harm of a treatment, given certain pre-determined operationalizations of benefit and harm and/or established thresholds for when prospect of net benefit (as defined in such-and-so a way) is too low to justify continued treatment.

### Rationing approaches

The third approach to determining when to end treatment for a patient is the rationing approach: determinations of whether treatment ought to be withdrawn are based on whether the likely outcomes from treatment are justifiable according to their cost (ie., whether they are cost-effective). AI could assist in making these determinations. For example, an AI model could inform whether the cost of chemotherapy for a particular patient’s disease stage can be justified given the likelihood of surviving a certain period of time (e.g., X number of months) with a sufficient level of quality of life. Although to our knowledge, there are no AI models that can simultaneously evaluate both cost predictions and effectiveness, some studies have found that AI can predict healthcare costs more efficiently and quickly in comparison to standard statistical modelling. For instance, some researchers have developed a deep-learning model using routine clinical notes as a dataset to accurately predict diagnosis-related group payment costs at a population level [43]. Another research team developed a linear regression analysis in machine learning models that could predict, with a maximum accuracy of 97.89%, overall healthcare costs incurred due to obesity [44]. Japanese researchers have also developed a machine learning model using national screening program data to accurately predict healthcare costs of high-need high-cost patients [45]. Given high-need, high-cost patients (those that account for top 5% of annual healthcare costs) require up to half of all healthcare costs – cost-effectiveness modelling could be of significant use to healthcare allocation measures and consequently, more accurately achieving predetermined distribution goals.

One approach to decision-making would be to develop an AI tool that measures the cost (or duration of resource use) of the treatment against the likelihood of treatment success as a single index and rank the outcome against other patients.^5^ One way to do this may be to rank the patients on healthcare costs, and separately on likelihood of success, and multiply these ranked positions to produce a single index. Wilkinson et al. [46] have suggested a metric based on the standard cost of the treatment measured against the predicted quality-adjusted life years (QALY) if the treatment is provided. Separate from financial cost, some treatments may need to be rationed because of scarcity, namely, when there are more patients in need of treatment than are able to be supported e.g., intensive care beds, extra-corporeal membrane oxygenation, or organs for transplantation. A similar ranking process, based on factors that are judged to be relevant to allocation of the treatment, could identify the relative priority of an individual patient.

Notably, as with quantitative outputs, AI-assisted rationing models cannot address the fundamental normative question concerning the most appropriate, just, or fair way to allocate healthcare resources. Such models require a prior determination of ethically defensible rationing. Alternatively, perhaps, models could provide a multi-modal output delineating different determinations according to different distributive standards, enabling decision-makers to decide between those different standards in light of their results. Either way, rationing decisions would still ultimately need to be made by people with relevant authority.

Regardless, further research, development, and investment into tools that more effectively and consistently measure the cost-effectiveness of treatment would provide better empirical bases for practitioners to determine the ethics of withdrawing disputed treatments. A distinct potential advantage of using explainable AI to identify whether treatment would be a justified use of limited resources is that it can help make transparent the basis for futility determination, as well as point to the appropriateness (or not) of seeking disputed treatment from other institutions.

### Process-based approaches

The fourth way to determine when to withdraw patient treatment is process-based approaches. These approaches focus on implementing proactive measures to avoid unnecessary disputes over the futility of treatment between patients/families and doctors, and/or providing guidance on how to resolve disputes once they have arisen. Some methods for resolving disputes govern the order of actions that must be taken if a dispute arises. Decision-tree algorithms might assist in these approaches through enhancing online dispute resolution tools [1]. Although often researched within legal contexts, the use of AI within online tools has been proposed as an effective means for efficiently resolving conflict with transparent and medical-specific processes that empowers and equips patients to self-advocate [47]. There are several levels of integration for which AI could be considered useful here.

First, AI could assist as an online tool that improves efficiency, as well as access for generally disempowered groups or populations, by completing administrative tasks [48]. We have already considered one tool that can undertake administrative tasks such as identifying the common and opposing positions of patients and doctors, identifying the key points of contention, and factual errors. However, AI-enhanced prognostic or diagnostic tools could also ensure that the prognostic information provided by doctors is reliable, which would protect patients in futility disputes from medical unnecessary overstatements. Using AI in this way could reduce the number of unnecessary disputes that arise from patients incorrectly assuming treatment would be beneficial to them, and doctors overstating their confidence in the likelihood of treatment outcomes and thus withdrawing or withholding treatment that could possibly reasonably be valued or beneficial to patients.

Second, AI could assist disputes over the subjective value of treatment in a substitutive capacity, possibly providing a first-instance step in the dispute resolution process prior to escalating disputes to committees, tribunals, or courts. For example, a family law AI tool, “Family Winner”, asks each party in a divorce conflict to nominally prioritize the items in dispute based on subjective value, algorithms to optimize the distribution of goods, and if this solution is rejected, it allows parties to rank the items still in dispute, and then prioritize the highest-ranked item of each party [49]. However, the use of AI to quantify subjective values has been criticized given dispute resolution processes require identification and acknowledgement of human emotions and underlying values: AI arguably fails to address the subjective emotions by representing human values as mathematical processes [31]. One study found that given healthcare disputes are often emotionally charged and the association between the influence of emotional intelligence and a propensity for a participant to modify their claims, AI-enhanced tools may only be well suited to patients with high emotional intelligence [50]. As such, other AI dispute resolution tools do not try to generate a solution amenable to all values. For example, ReConsider is an AI-enhanced online dispute resolution tool that asynchronously assists parties to understand and restructure their positions until an agreement is made, or the parties acknowledge there is no means to agree, in which the system draws inferences akin to a judicial party if the matter was litigated [51]. Some research has predicted that given the prevalence of Big Data in healthcare, such uses will be quickly adopted in medical disputes [52].

Third, AI informed by community preferences and data might assist in disputes by making determinations about the normative reasonableness of providing the treatment. For instance, a hospital might survey its local community on particular values, preferences, and trade-offs to determine whether there is consensus on when certain treatments are futile or when institutions are justified in withdrawing or withholding medical treatment. The hospital may find that there is consensus in some cases, but sustained disagreement in others. The hospital may then integrate this data into an AI model that maps real-time futility disputes between patients and doctors onto local data through a predictive algorithm to provide an answer as to whether the disagreement in question is reasonable by comparing the views of the patient and medical team with views determined by public consultation. Public consultation on end-of-life preferences could assist medical teams in determining when the patient is being reasonable in expecting certain outcomes [19]. That is, public consensus will show the extent of the public’s wishes on end-of-life care, and how prevalent such wishes are within the community [53]. How to draw a line between unreasonable and reasonable disagreement is normatively difficult, and AI might empower doctors to make transparent and empirically-backed determinations of reasonableness by explaining how the patient’s values measure against community standards and variation [54–56].

## Should we use AI to help?

We have identified potential ways in which existing or emerging AI could potentially improve decision-making in cases of disputed treatments. However, there is a further question of whether we ought to use AI to assist with these kinds of decisions. Given the increasing scholarship on the justifiability of using AI in medical decision-making, we will not reiterate general concerns with the use of AI in such contexts. These have been sufficiently engaged and explored elsewhere: a comprehensive treatment falls outside the scope of this paper.^6^ Instead, we will consider and evaluate concerns that we deem to be unique to the use of AI in medical futility cases.

First, it is important to note that we are not suggesting deferring decisions about futility to AI. AI should be seen as a decision aid to facilitate communication, deliberation, and reflection; it should assist decision making by patients, doctors, and ethics committees. But ultimately doctors or courts must retain the final responsibility for such life and death decisions, and they must take all relevant factors into consideration, which may include the output from AI.^7^ However, we acknowledge the worry that the use of AI might introduce further complications or issues into determinations about futility. Algorithmic bias, for instance, might arise: doctors may be tempted to defer decisions to AI without incorporating their medical and human judgement into medical decisions [67]. Incorporating the use of AI into medical decision-making is not without challenges. For the purposes of this paper, however, we are concerned with the specific challenges of incorporating the use of AI into decisions about medical futility.

### Accuracy

Prediction accuracy is a general concern with AI tools but is critically relevant to futility determinations for two reasons. First, the consequences of a declaration of futility are that the patient may have the treatment withheld or withdrawn. For life-sustaining treatment, this will potentially lead to the patient’s death: given the gravity of such consequences, such decisions ought to be as certain and reliable as reasonably possible. Second, if the reasons for which doctors are arguing the treatment is futile are uncertain, then it is more difficult to determine whether the patient’s preferences for treatment are reasonable. The more unreliable and uncertain the medical predictions are, the more scope there is for dispute between patients and doctors as to the reasonability of the benefit of treatment.

As such, there may be concerns that if AI is to be used in making determinations of patient preferences, medical predictions, or cost-effectiveness, it ought to be at least more accurate than current decision-making standards. Certainly, if any of the elements of AI prediction result in less accurate clinical predictions than are currently made clinicians, there are good reasons not to use them. This requirement may result in reduced ability to apply AI tools outside the specific community and health care context in which they have been developed. This concern is compounded by the fact that the significant majority of data that is used to train medical AI is predominantly English-language data originating from clinical contexts in the United States.^8^ However, the clinical meaningfulness, availability of therapeutic technology, and digital salience is not context independent. Consequently, medical AI is inherently and deeply conditioned by context-specific medical infrastructures, cultural frameworks, and socioeconomic structures from which such data is drawn. To this extent, ensuring implemented AI tools are proportionally trained on local data and values relevant to that context, and then validated in local communities may be necessary to ensure such devices meet accuracy standards.

One important consideration here is that humans are not always accurate: research suggests that clinical prognoses, particularly in palliative care, are often inaccurate [68], and that clinicians struggle with prognostic determinations, particularly within the end-of-life context [69]. If, therefore, an AI tool is consistently shown to be more accurate than humans in determining patient prognostics, then there might be a reason to prefer the inclusion of AI as to improve the certainty of empirical claims within futility disputes [70]. Whilst we acknowledge that clinical accuracy is different from model accuracy, given human users could misinterpret, misapply, or simply mistrust outputs, the integration of AI into futility determination can be regulated and measured during a period of real-world prospective validation to demonstrate practical effectiveness [71]. One challenging normative question is what degree of accuracy is required for treatment to be withdrawn, but arguably, the threshold ought to be at least better than current human standards.

A further challenge may arise if AI is to make quantitative determinations of futility an produces a different prediction to the doctor. This may result in a binary disagreement becoming tripartite, in which the patient, doctor, and AI all have competing positions or views over a determination of medical futility. This may also arise if AI is used within the process-based approaches, in ways akin to judicial judgments. This risk of complication may further burden hospitals and medical teams seeking to resolve already complex disagreements.

How we deal with this additional layer of disagreement will likely depend on the overall approach taken. If an institution primarily prioritizes medical opinion for determinations of futility, then the AI model with greater proven accuracy might carry the most weight in making determinations of futility (it may support the medical perspective). However, in institutions that prioritise patient preferences, an alternative AI prognosis may bear little on determinations of futility. Rather, if the more accurate AI prediction indicates low prognostic survivability or quality of life, such a result might be discussed with the patient as a reason for them to reconsider their request for further treatment. It remains to be seen how this will alter the dynamic, but there is at least the potential that introducing additional factors or views to this binary dispute may lead to increased resolutions or avoided disagreements, despite the possibility of some cases becoming more complicated.

### Self-fulfilling prophecies

In futility disputes, there is a concern that determining that treatment is ‘futile’ is potentially a self-fulfilling prophecy: once treatment is considered futile, any data would likely show that the patient did indeed die upon the withdrawal of treatment and thus proving that treatment was futile. Whilst this is already a concern with decisions about the futility of treatment, the use of AI to make such determinations raises concerns that this problem might be exacerbated through the machine learning process in which the machine learns from the outcomes of its own decisions. The more widespread the use of AI in predictions and decision-making, the more pervasive the potential influence on outcomes.

This, although legitimate, concern may be partially abated by discounting outcomes that have been influenced by previous decisions i.e., to give them less statistical weight compared with outcomes that have not been influenced. Whilst this might limit the machine’s capacity to learn by limiting its data pool, preventing the use of self-fulfilling data means that AI decisions might be only as problematic as self-fulfilling clinical predictions. Furthermore, when AI is able to draw on large data sets, it may be possible to distinguish decisions that did not have access to AI-based predictions, or where treatment was provided notwithstanding predictions of poor outcomes. Mitigating the negative impacts of self-fulfilling machine learning would require proactive regulation in the AI design process.

### Bias

Much literature has expressed concerns of using AI in medical decision-making given its reliance on big data, and the ways in which medical data has historically been biased [58, 67, 72–74]. Similarly, there are many ways in which cost-effectiveness, as a measurement of fairness, has been criticized as being discriminatory; it will be discriminatory against older persons (ageist), disabled people (ableist), women (sexist), and persons of colour (racist) [75]. Cost-effectiveness predictions, for instance, will also be sexist if the training data reveal a different life expectancy or different quality-of-life benefit between the sexes due to socioeconomic, rather than physiological, reasons. As such, a concern with using cost-effectiveness predictions is that such models amplify bias in data sets causing unfair results. Given disadvantaged groups often require more healthcare to treat their conditions owing to unfair systematic socioeconomic inequalities that undermine their health, cost-effectiveness predictions might unfairly predict that disadvantaged patients with higher costs will be less cost-effective to treat in comparison to patients with similar conditions [76]. If the principle of distributive justice is being used to defend the withdrawal of treatment, we ought to be concerned that cost-effectiveness is not sufficient to justify this decision but consider equity, equality, and fairness.

Whilst these are legitimate concerns that apply broadly across healthcare prediction systems, within the context of determinations of futility, they may be at least partially mitigated where the AI system replaces standard group-based predictions that classify patients by disease type with individual demographic data, which may reduce the risk of systematic bias against individuals on the basis of gender, race, ability, or age. In such cases, healthcare institutions may face a trade-off between individual-level predictive accuracy and broader considerations of fairness and justice [77]. Indeed, Kleinberg et al. [78] have shown that we cannot achieve calibration between and within groups, and at the same time, balance false negatives and false positives between groups: normative trade-offs are an inevitable decision rather than an introduced factor due to the mere fact that health and healthcare outcomes are different between various populations.

However, group-based approaches will not necessarily eliminate systematic bias at the population level. Conditions that disproportionately affect disadvantaged groups may be more costly to treat precisely because of historical underinvestment in researching those conditions, resulting in fewer and more expensive treatment options for such groups. It is worth noting, however, that this is not a problem created by AI; it reflects longstanding structural inequities in healthcare research and funding that exist independently of any algorithmic system.

Whilst we must actively and transparently make such normative decisions, we also ought to consider the advantages of using AI in combatting bias in current futility decisions. Currently, as cited above, many resource-based futility determinations are being made without systematic data but rather based on individual judgment, which is highly prone to bias [79]. As such, even with training data biases, AI models may be less prone to bias than human decision-makers [79]. Some research suggests that it may be possible to use AI (either the same system, or a complementary system) to interrogate decisions for the influence of bias in determinations [80, 81]. This is considerably easier than with human decisions, particularly when humans unconsciously hold biases.

Furthermore, developers can explicitly exclude certain factors from decision-making, for instance, they can exclude the use of race or gender from prognostic predictions. This approach warrants some caution, given there are proxies for these factors, e.g., a particular health condition as proxy for race [82], and these proxies cannot always be identified: they are unpredictable and can sometimes overlap. Eliminating factors that directly cause bias (e.g., such as race) from algorithmic decision-making will therefore not automatically lead to the elimination of bias. As such, some research is considering how to address both explicit and proxy bias, such as through ensuring AI tools are explainable and transparent about the use of bias and proxy bias to its users, particularly so that regulators and doctors can normatively evaluate the AI decision for harmful biases [83].

### Black-box and transparency

Another concern with using AI in futility decisions arises when AI systems are unexplainable: they are black-box algorithms. The black box describes the way in which some AI tools cannot explain the exact pathway from input to output, and the weight given to each factor that resulted in the prediction or recommendation. This is a general issue with many AI tools, however, it is uniquely concerning to futility decision-making because research consistently shows that a key contributing factor to futility disputes is a lack of transparency about medical expectations and the principal reason for withdrawal i.e., futility versus rationing [3]. Given the importance of transparency in disputed treatment decision-making, uninterpretable and unexplainable black-box AI may be poorly suited to achieving these goals.

One response we might make here is that when these AI tools produce predictions or recommendations with greater accuracy than current methods, this improved accuracy may offset these downsides of non-transparent decisions. In situations where we have competing decision-makers (human versus AI) to produce the same outcome (e.g., prognosis), we ought to consider the strengths and weaknesses in achieving various goals and medical values. Human decision-makers might be more transparent than black-box AI because they can give explanations for their decisions; however they might have statistically lower accuracy in diagnosing or predicting patient conditions and outcomes. In contrast, AI decisions might be less transparent than humans because they cannot give explanations about how they arrived at a decision but might have greater accuracy in making diagnostic or prognostic decisions thus resulting in better healthcare outcomes. That is, we must choose between prioritising transparency and beneficence, and it is not so clear that we ought to always prioritise transparency. For example, if greater accuracy enables patients to make more autonomous decisions (better aligned with patient values), then such benefits of a non-transparent decision may outweigh concerns about a lack of transparency [63]. The use of AI systems ought not to be necessarily rejected for merely being unexplainable; transparency is but one relevant factor in determining the justifiability of a determination of futility [84].

Furthermore, clinical decisions are often also ‘black box’ [79, 85, 86]. A clinician may not be able to explicitly answer why they have arrived at a judgement to recommend a certain treatment and may also merely provide post-hoc rationales for their decisions. The imperative to produce explainable AI is not absolute, particularly if other AI tools or doctors can provide post-hoc rationales for black-box AI decisions. Nevertheless, some models have already proven to be both explainable and accurate at predicting mortality rates in critically ill patients under mechanical ventilation [40]. Accurate and explainable AI is justifiably more useful and ethical than merely accurate AI, but we ought to be careful of being overly cautious of the use of AI for merely having a black box.

### Recommendations for use of AI in futility determinations

In addition to the concerns we have raised above, there are other general concerns arising from the use of AI in healthcare [87], particularly pertaining to privacy and confidentiality of patient data, regulation of AI devices, and review and procedural fairness [88–90]. Consequently, whilst we do not seek to provide an all-things-considered judgement of the justifiability of using AI to make determinations of medical futility, we believe there should be context specific guidance. Comprehensive guidance would require further analysis beyond the scope of this paper, but in this space we can make some brief recommendations for future research, regulation, and implementation of such devices.

First, the presence of ethical risks, such as inaccuracies, self-fulfilling prophecies, bias, and black-box models, is not sufficient to render the use of AI unjustified. We can have some degree of risk tolerance in deployment of AI for futility determinations. There are risks with any approach; not all harms are equally significant, and not all of these concerns will be present in each context. The risks and harms arising in each context must be identified and mapped according to the particular AI model used, the purpose for which it is used, and the existing practice or standard already in place. For instance, if an AI system demonstrates meaningfully greater capability to predict patient preferences than existing standards for a particular patient population within surrogate decision-making, then even if such models are not equally effective at predicting all people’s preferences, or even if they lack explainability, such utility may be sufficient to justify their use.

Second, accuracy is a necessary condition for the use of AI in determinations of medical futility [87]. In all stages of determinations of medical futility – patient preferences, medical diagnostics and prognostics, healthcare distribution decisions, and process-based techniques – due to its additional risks and harms, AI needs to be at least as accurate as alternatives. However, accuracy is not sufficient to justify its use. There are independent moral constraints on the justifiability of using AI in determinations of medical futility. If, for instance, research indicates patients generally prefer to have appointed surrogates make decisions on their behalf rather than AI, even knowing they are less accurate, respecting patient autonomy may preclude their use, or preclude sole deference to AI without involving family.

Third, not all AI contributions ought to be considered determinative of futility decisions. For instance, even if a hospital adopts a patient-centered approach to resolving futility disputes, that a P4 model (or equivalent) suggests that a patient would want a particular treatment does not mean that this particular preference must be followed. Similarly, if a hospital adopts a medical approach to resolving futility disputes this does not mean that an AI-generated prognosis ought to be considered determinative of the final decision. AI predictions, whether about patient preferences or prognostic outcomes, can be used in facilitation or conjunction with existing approaches. AI tools might relieve doctors of the burden in having to explain prognosis to patients, the accuracy of the prognosis, or any limitations pertaining to prognostic models, particularly in ways that are accessible and appropriate for the family. AI tools might constructively facilitate discussions rather than be determinative of treatment.

Fourth, any integration of AI into healthcare must be proactively regulated, validated, and tested. This includes using policy and law to govern the ways AI impacts patient care and autonomy, such as through data privacy and confidentiality, ensuring human oversight, validating devices against industry and professional standards, and implementing requirements that AI models be adequately informed by local data [53, 87]. Furthermore, given this paper is largely theoretical, AI devices should be clinically validated through testing for accuracy relative to their intended purpose (e.g., patient prediction or diagnosis), and to ensure devices are comparably more accurate and efficient at reducing futility disagreements. The introduction of such devices should be accompanied by education, training, and legal protection for healthcare professionals who use them. Finally, AI devices should not be used in ways that significantly increase healthcare professional workloads [91].

## Conclusions

The difficulties in determining when treatment becomes allegedly futile and resolving disputes over the alleged futility of proposed treatment are costly, burdensome, and currently inconsistently addressed in both practice and the literature. We propose that current and future advancements in AI technology provide an innovative and likely useful tool in both assisting determinations of when treatment becomes futile, and how to proceed when such determinations are made when patients or families disagree. Realising such benefits will require new research in normative and empirical bioethics, as well as in computational and AI development. We have proposed using AI as a tool to support existing approaches to futility determination. However, AI cannot, itself resolve the question of the correct approach. Given the concerns of using AI in futility determinations, we suggest that its development and use ought to be carefully regulated. Nevertheless, in this difficult area of medical ethical decision-making, AI may have an important role to play.

## Limitations

There are some limitations to narrative reviews, such as questions around bias and rigor due to the subjective nature of the review. We attempted to reduce these limitations by not favouring a single conceptual framework for understanding medical futility, as well as critically analysing whether AI should be used to address these issues, rather than simply assuming that it can, and therefore we ought to use it. A further limitation is that the use of AI within futility determinations is largely nascent and theoretical, and so this review largely anticipates potential challenges and use cases rather than analysing existing evidence.

## Data Availability

No datasets were generated or analysed during the current study.

## Footnotes

1 The financial cost of providing futile care is difficult to determine given the inconsistencies in definitions and criteria. However, one Australian study found that futile care was provided in 12% of end-of-life admissions, costing $A12.4 million for three hospitals over 6 months. The mean length of stay after treatment was judged to be futile was 15 days (including 5 days in intensive care). The authors estimating an annual national cost of $A153.1 million AUD expended on futile treatment [7]. A Canadian study found that providing futile treatment to 12 patients over the course of at least 4 months cost almost $8 million CAD [8]. An American study found that providing futile treatment for 3.4% of elders incurred 8.9% of reported costs of Medicare patients admitted in 171 Oregon and Washington hospitals in 2001 to 2002 [9].

2 We acknowledge that various terms have been proposed instead of futility: non-beneficial treatment [14]; potentially inappropriate treatment [15]; perceived inappropriate treatment [16]; inappropriate or disputed treatment; medically ineffective interventions [17]; and, medically inappropriate treatment [1]. There has been little agreement on both the name and conceptual definition of futility, and we shall address this properly below.

3 This term was originally termed by Schneirdamn et al. [18] and referred to whether the treatment had not worked in the previous 100 similar cases.

4 There is one, intriguing exception, where there are predicted contradictory or paradoxical preferences (e.g., when the AI predicts that the patient would give no weight to AI-mediated patient preferences).

5 We acknowledge that this is more complicated when various funders are contributing to payment e.g., through insurance, by the patient themselves, or by other bodies.

6 Some of the general concerns with using AI in healthcare are that it could reinforce and exacerbate harmful biases against certain patient groups [57], that black-box AI is harmful insofar as it is unexplainable and nontransparent [58], concerns that doctors might be swayed by algorithmic bias and adopt AI recommendations without human analysis [59], worries that AI cannot meet ethical codes of conduct requirements [60], concerns that AI is not equipped to make moral or emotional decisions [61], and worries that the use of AI undermines patient autonomy and capacity to consent to treatment [62, 63].

7 Some research suggests that clinical output from AI decision-making can sometimes make human decisions less accurate. For example, research shows that sometimes, human-AI made decisions cause performance loss in comparison to humans or AI alone [64] and that sometimes doctors diagnostic accuracy is worse when relying on inaccurate advice from ‘AI’ (human advice labelled as AI to participants) [65]. However, other research shows that this is not necessarily always the case, and that these findings highlight how research ought to be undertaken to understand the conditions that support complementarity between humans and AI to ensure improved performance [66]. This includes understanding *when* and at what stage in decision-making AI ought to be included, the amount of information provided to the human to avoid cognitive overload, and when reliance strategies are ineffective.

8 We thank our anonymous reviewer for raising this point.
